# Relationship between Smoking and Acute Mountain Sickness: A Meta-Analysis of Observational Studies

**DOI:** 10.1155/2017/1409656

**Published:** 2017-11-12

**Authors:** Cristina Masuet-Aumatell, Alba Sánchez-Mascuñano, Fernando Agüero Santangelo, Sergio Morchón Ramos, Josep Maria Ramon-Torrell

**Affiliations:** Bellvitge Biomedical Research Institute (IDIBELL), Preventive Medicine Department, University Hospital of Bellvitge, L'Hospitalet de Llobregat, Catalonia, Spain

## Abstract

**Aims:**

Previous epidemiological investigations of the relationship between smoking and acute mountain sickness (AMS) risk yielded inconsistent findings. Therefore, a meta-analysis of observational studies was performed to determine whether smoking is related to the development of AMS.

**Methods:**

Searches were performed on PubMed, Scopus, Embase, and Web of Science for relevant studies that were published before November 2016 reporting smoking prevalence and AMS. Two evaluators independently selected studies, extracted data, and assessed study quality. The pooled relative risks (RRs) and 95% confidence intervals (CIs) were obtained using random-effects models. Subgroup analyses were performed according to the type of participant, altitude, and study design.

**Results:**

A total of 11 observational studies involving 7,106 participants, 2,408 of which had AMS, were eligible for inclusion in this meta-analysis. The summary RR for AMS comparing smokers to nonsmokers was 1.02 (95% CI: 0.83 to 1.26). Specific analyses for altitude, type of participant, and study design yielded similar results. There was significant heterogeneity for all studies (*Q* = 37.43; *P* < 0.001; *I*^2^ = 73%, 95% CI: 51% to 85%). No publication bias was observed (Egger's test: *P* = 0.548, Begg's test: *P* = 0.418).

**Conclusions:**

The meta-analysis indicates that no difference was found in AMS risk with regard to smoking status.

## 1. Introduction

Climbers, trekkers, workers, and tourists who travel to high-altitude destinations are at risk of altitude illness due to hypoxia. Altitude illness, which includes acute mountain sickness (AMS), high-altitude cerebral edema (HACE), and high-altitude pulmonary edema (HAPE), may occur in any traveller who reaches 2,500 meters above sea level (MASL) or higher if inadequate acclimatisation occurs [1]. Millions of people travel to typical high-altitude destinations annually, such as Cuzco (3400 MASL, Peru), La Paz (3780 MASL, Bolivia), Lhasa (3660 MASL, Tibet), Everest Base Camp (5364 MASL, Nepal), and Kilimanjaro (5895 MASL, Tanzania).

The most common form of altitude illness is AMS, which generally resolves within 24–72 hours of acclimatisation. The incidence of AMS varies from 25% to 75% [1] in the general population but is unknown in smokers. AMS risk is affected by a traveller's characteristics such as genetic traits [2], age [3], previous exposures to high altitude and experience [4], destinations, itinerary, rate of ascent, and exertion [5]. Alcohol intake avoidance [4], carbohydrate ingestion [6], water intake [7], and acetazolamide use [8] may prevent AMS. Training or physical fitness may not be significantly associated with AMS, but the influence of some covariates, such as obesity or smoking status, is debatable. High altitudes cause a decrease in barometric pressure and inspired oxygen pressure. Therefore, reduced alveolar oxygen pressure, oxygen arterial pressure, and oxygen arterial saturation rate occur, which greatly reduces oxygen availability into tissues.

Smoking tobacco increases the carbon monoxide (CO) concentrations in the airways and the blood. Carbon monoxide's haemoglobin-binding affinity is over 200 times that of oxygen. The level of CO is an indirect measure of blood carboxyhaemoglobin (COHb). The percentage of COHb is the proportion of red blood cells carrying CO instead of oxygen. The normal level of CO for a nonsmoker depends on background levels in the air but is usually lower than 5 ppm, and percent of COHb is lower than 1.43 [9]. The level of CO for a smoker is usually much higher according to the time of the day, the number of tobacco products smoked, and how the smoke is inhaled. The level of CO for a smoker of 20 cigarettes per day is usually around 20 ppm and percent of COHb higher than 3.83. Increased levels of carbon monoxide in the bloodstream have a negative ionotropic effect [10] and can limit the amount of oxygen transported in muscular capillaries, which adversely affects skeletal muscle performance. Smoking causes an increase in carboxyhaemoglobin levels, resulting in a leftward shift of the oxyhaemoglobin dissociation curve when carbon monoxide is present in the blood. Carbon monoxide reduces the formation of 2,3-DPG by inhibiting glycolysis in the erythrocyte. Nicotine stimulates the sympathetic nervous system, which can lead to increased levels of catecholamine, thereby increasing a person's heart rate and stroke volume [11]. The tar produced by the burning of tobacco can increase pulmonary airway resistance or reduce the contact surface area between oxygen and pulmonary capillaries, thereby decreasing the capacity of the arteries to transport oxygenated blood during exercise. Therefore, smokers exhibit a lower capacity to transport oxygen because of increased carboxyhaemoglobin and difficulties in breathing control and alterations in vascular tone, neurotransmission, and cellular metabolism due to carbon monoxide and lower vasodilatation in hypoxic environments than nonsmokers [11].

Previous epidemiological investigations of the relationship between smoking and acute mountain sickness (AMS) risk yielded inconsistent findings. Some studies identified smoking as a risk factor [12, 13] or a protective factor [11, 14], but other studies failed to show any significant association [4, 15–18]. However, only one study was specifically designed to study smoking as a risk factor for AMS [11]. Therefore, a meta-analysis of observational studies (cross-sectional studies, case-control or cohort studies) was performed to determine whether smoking was related to the development of AMS.

## 2. Materials and Methods 

### 2.1. Data Sources and Search Strategy

**Figure 1:**
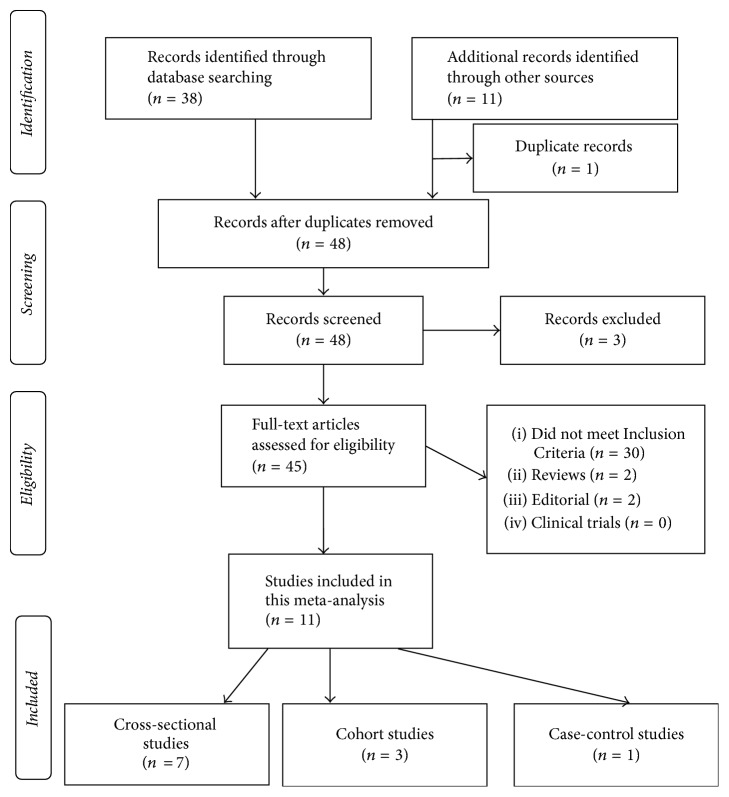
PRISMA flow chart of the included studies selection process.

The PRISMA statement [19] for the reporting of systematic reviews recommended by the Cochrane Collaboration was followed while conducting this meta-analysis (see Figure 1). Observational studies (cross-sectional studies, case-control, and cohort studies) on smoking and the risk of AMS were included in our meta-analysis, and language, publication status, or article type was not considered. Two investigators conducted a systematic literature search of the electronic databases PubMed (from 1965 to November 2016), SCOPUS (from 1965 to November 2014), Embase (from 1965 to November 2016), and Web of Science (from 1986 to November 2016). Searches were performed using the search terms under two search themes that were combined using the Boolean operator “AND.” For the theme of “AMS,” a combination of Medical Subject Headings (MeSHs), entry terms, and text words was used: “acute mountain sickness,” “altitude illness,” “mountain sickness,” “high-altitude cerebral edema,” and “HACE.” For the theme of “Smoking,” “smoking” and “tobacco” were used. In addition, all references cited in relevant original and review articles were searched manually.

### 2.2. Selection Criteria

Eligible studies met the following inclusion criteria: (1) the study was an observational study (cross-sectional study, case-control study, nested case-control study, or cohort study); (2) the exposure of smoking was described; (3) the outcome of interest was AMS; (4) the study reported the percentage of AMS according to tobacco exposure, the relative risk (RR) or odds ratio (OR), and the 95% confidence interval (CI) for the association between smoking and risk of AMS; and (5) a Newcastle-Ottawa Scale (NOS) or adapted Newcastle-Ottawa Scale (aNOS) score of 5 or greater indicated moderate- to high-quality studies [20]. Studies that did not document the frequency of AMS, animal experimentation studies, and mechanistic research studies were excluded. Studies in which the exposure of interest was not sufficiently explained were also excluded to avoid the combination of studies that were not comparable. Two investigators independently conducted the study selection.

### 2.3. Data Extraction and Quality Assessment

Two reviewers independently performed data extraction and quality assessment. The following information was extracted from each eligible study: first author's surname, year of publication, study location, study design, source of study population, type of exposure or population, sample size, number of events, proportion of smokers, definition of smoking, definition of AMS, estimated effect size (OR or RR), 95% CI, and covariates adjusted in statistical analyses. No studies reported several multivariable-adjusted effect estimates based on smoking behaviour. Therefore, a result that was fully adjusted for potential confounding variables was not selected. Quality assessment was conducted using the nine-star Newcastle-Ottawa Scale (NOS); see Table 1. We considered studies with an NOS score of 5 or greater to be moderate- to high-quality studies [20]. After data extraction and assessment, the information was examined and adjudicated independently by an investigator who referred to the original articles.

### 2.4. Statistical Analysis

The relationship between smoking and risk of AMS was examined using the OR or RR and 95% CI in each study or the frequency of AMS in smokers versus nonsmokers. Prevalence ratios (PRs) in the cross-sectional studies and the OR in case-control studies approximated the RR because the absolute risk of AMS is low [21]. The AMS detected was the incidence of AMS in all cross-sectional studies because these studies asked about the appearance of AMS when the subjects reached the altitude where the study was conducted. The incidence of AMS and its 95% CI was described depending on smoking status and altitude. Therefore, the PRs and ORs should likely be considered RRs in these studies. A meta-analysis comparing the risk of AMS between smokers and nonsmokers in all included studies was performed. Smokers were defined as subjects who smoked 10 or more cigarettes per day currently. Mountaineers were defined as trekkers or climbers who were physically fit persons, who knew the rules of high-altitude climbing, and were aware of the importance of acclimatisation. AMS was defined as an Environmental Symptom Questionnaire (ESQ) score greater than 0.7 [22]; Lake Louise score (LLS) greater than or equal to 3 [23]; or using a Study-Specific Questionnaire (SSQ) [12, 15, 24]. If a single study reported results for different populations (e.g., different altitudes or destinations) but did not report the overall results, the results for each population were calculated as a separate study [25]. The ESQ has 67 questions, evaluates the presence and severity of AMS symptoms (headache, nausea, and the general feeling of ill health), and determines whether an individual has no AMS (ESQ < 0.7) or AMS (ESQ ≥ 0.7). The ESQ contains many superfluous questions, requires multiplying each response by a factorial weight, and has been criticised because it is validated against the simple item, “I feel sick.” The LLS has five questions, evaluates the presence and severity of AMS symptoms (headache, gastrointestinal symptoms, fatigue and weakness, dizziness and light-headedness, and difficulty sleeping), and determines whether an individual has no AMS (LLS < 3), mild AMS (LLS 3–5), or severe AMS (LLS > 5). The LLS has been validated against clinical assessment. Some studies show that for criterion scores yielding similar prevalence of AMS, ESQ labels 20% of cases differently when compared to LLS. The SSQ means that each study used a specific questionnaire as a translation of a Chinese scoring system for AMS [15, 24], which presents a high correlation to LLS (*r* = 0.820) or restricted the case definition of AMS to those persons who were deemed ill enough to undergo compression chamber treatment [12]. Reported incidences and identifiable predictive factors of AMS [26] depend on study design (RCTs in comparison to cohort and cross-sectional studies). Subsequently, we conducted subanalyses by epidemiological study design (cross-sectional studies and case-control studies versus cohort studies), altitude (high altitude defined as <3500 MASL versus very high altitude defined as ≥3500 MASL) [11], type of participant characteristics (mountaineers versus nonmountaineers), and NOS (≥6 versus <6) as a sensitivity analysis. According to inclusion criteria, NOS < 6 refers to NOS = 5. All included studies presented different quality assessment scores. Therefore, we performed sensitivity analyses according to NOS score and designated NOS scores from 5 to 6 as moderate and NOS scores equal to or greater than 7 as high. A random-effects model was used to estimate the pooled RRs with 95% CIs because there was evidence of heterogeneity [27]. Forest plots were used to assess the RR estimates and corresponding 95% CIs visually. We could not perform a two-stage, random effect, dose–response meta-analysis to examine the potential nonlinear relationship between smoking dose and risk of AMS because smoking dose was not reported in most studies. Heterogeneity between studies was evaluated using the Cochran's *Q* and *I*^2^ statistics [28, 29]. The probability of publication bias was assessed using the Egger regression test [30] and Begg's funnel plot [31]. We evaluated the effect of publication bias using the trim and fill method [32]. Stata version 12.0 software (Stata Corporation, College Station, TX) was used for all analyses, and all statistical tests were two-sided. *P* < 0.05 was considered statistically significant.

## 3. Results 

### 3.1. Description of the Selected Studies

Forty-nine records were retrieved using the specified search strategy in November 2016. After reading the titles and abstracts, 4 records were excluded, and 45 studies were retained for further evaluation by a reading of the full text. Thirty-four studies did not meet the inclusion criteria or were not an original study. Finally, we identified 11 full-text studies on smoking and AMS for inclusion in the meta-analysis, including 7 cross-sectional studies [4, 13, 16–18], 3 cohort studies [11, 14, 15], and 1 case-control study [12]. Figure 1 depicts the search process. These 11 studies included 7,106 participants and 2,408 AMS cases (5 studies in Asia; 5 studies in Europe; and 1 study in America).  Table 2 presents the general characteristics of the included studies. Studies asked the same fundamental question about risk factors linked to AMS, but the populations (mountaineers, tourists, workers, and military troops), study design (cross-sectional studies, nested case-control study in an occupational cohort, and cohort studies), AMS definition, and smoking status definition were not identical. Therefore, the studies varied in specific ways.

### 3.2. Description of the AMS Incidence

The overall incidence of AMS in the studied population was 25.02% (95% CI, 24.01 to 26.03); when they climbed to altitudes between 2500 MASL and 3500 MASL, it was 19.61% (95% CI, 16.16 to 23.06); higher than 3500 MASL, it was 25.44% (95% CI, 24.39 to 26.49); if they were climbers or mountaineers, it was 34.31% (95% CI, 32.50 to 36.12); and, in nonmountaineers, it was 19.54% (95% CI, 18.38 to 20.70).

### 3.3. Smoking and Risk of AMS

**Figure 2:**
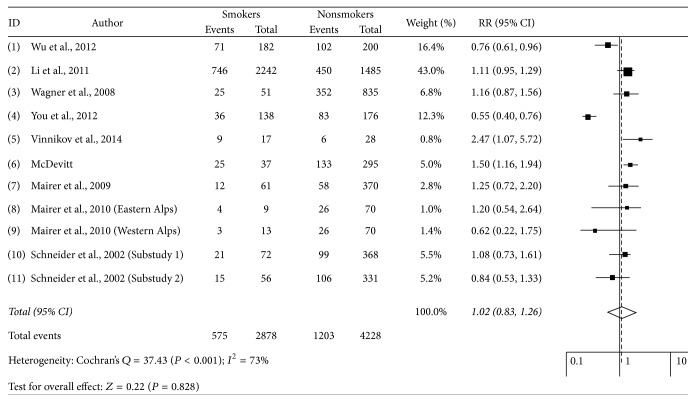
Forests plots of relative risks of AMS and smoking. AMS: acute mountain sickness; CI: confidence interval; RR: relative risks. The relative risks were obtained by random effect (DerSimonian and Laird).

Figure 2 shows the summary RR of AMS for smoking status. The summary RR for smokers was 1.02 (95% CI, 0.83 to 1.26) for AMS, with some heterogeneity (*I*^2^   = 73.0%, *P* < 0.001). The Begg's funnel plot did not show any asymmetry (*P* = 0.418), and Egger's test revealed no publication bias (*P* = 0.548).

### 3.4. Subgroup Analysis

Table 3 shows the results of subgroup analyses according to study design, altitude, quality assessment of the studies, and type of participant. The reported RR always was considered smoking over nonsmoking. Restriction of analysis to cohort studies revealed a summary RR of AMS for smoking status of 0.79 (95% CI, 0.54 to 1.17) with high heterogeneity (*I*^2^ = 89.0%, *P* < 0.001). Two cohort studies showed a statistically protective effect of smoking on AMS development. The RR for AMS risk for case-control or cross-sectional studies was 1.20 (95% CI, 0.99 to 1.47) without heterogeneity (*I*^2^ = 32.0%, *P* = 0.169). Two of these studies showed a significant risk effect of smoking on AMS development. Stratification by altitude revealed an RR for AMS of 1.24 (95% CI, 0.78 to 1.95) without heterogeneity (*I*^2^ = 25.0%, *P* = 0.922) for altitudes below 3500 MASL and an RR of 1.00 (95% CI, 0.79 to 1.26) with some heterogeneity (*I*^2^ = 78.0%, *P* < 0.001) for altitudes equal to or above 3500 MASL. However, controversial association results were present. The RR for AMS according to type of exposure or population was 1.17 (95% CI, 0.97 to 1.41) without heterogeneity (*I*^2^ = 23.0%, *P* = 0.253) for mountaineers or in studies with moderate quality (NOS = 5 or aNOS = 5) and 0.92 (95% CI, 0.62 to 1.36) with high heterogeneity (*I*^2^ = 87.0%, *P* < 0.001) for nonmountaineers (travellers, workers, or military troops) or in studies of high quality (NOS ≥ 6 or aNOS ≥ 6).

### 3.5. Sensitivity Analysis

Table 4 shows the results of sensitivity analyses. The sensitivity analysis removed one study at a time to assess the robustness of the overall results. Sensitivity analyses and subgroup analyses results were consistent with the overall results, which indicated that there was no heterogeneity from study design, study quality, altitude, or type of participants. However, one study [14] decreased statistically significant the heterogeneity to 59%, probably because it is a military cohort study conducted in China at an altitude over 4300 MASL, and the AMS definition was LLS ≥ 4.

## 4. Discussion

The meta-analysis indicates that no difference was found in AMS risk with regard to smoking status (RR 1.02, 95% CI, 0.83 to 1.26) independent of altitude, training, or type of questionnaire used.

### 4.1. Smoking

Smoking status is thought to participate in acute mountain sickness, and it is considered a traditional risk factor for the development of AMS [1, 11]. However, some epidemiological studies that evaluated the development of AMS did not describe smoking as a risk factor, and most epidemiological studies were not specifically designed to evaluate the relationship between smoking and AMS. These studies describe controversial results because negative and positive associations are reported. This lack of agreement between the results of previous studies may be due to differences in altitude, study population, epidemiological designs, or residual confounding. Previous studies were systemically reviewed to explore the association between smoking and the risk of AMS because of the importance of this possible association in clinical practice and public health. To our knowledge, prior to our study, two meta-analyses explored the association between smoking and AMS [33, 34] with different results. Vinnikov et al., 2016, found that smoking was not significantly associated with AMS (OR 0.88, 95% CI, 0.74 to 1.05) whereas Xu et al. [34] found that smoking may protect against AMS development (OR 0.71, 95% CI, 0.52 to 0.96). Different selection criteria may lead to different results; we used strict inclusion criteria, high-quality studies, and control for confounding variables and heterogeneity by different methodological tools. Our meta-analysis results contrast with one meta-analysis [34] but accord with the other one [33]. Meta-analysis of 11 observational studies suggested an AMS incidence rate of 25.02%, being lower if the traveller climbed below 3500 MASL than if the traveller climbed higher than 3500 MASL. This probably reflects comparison between climbers, who go to higher altitudes, and nonclimbers. Meta-analysis suggested that smoking was not significantly associated with AMS risk. The association was not significantly affected by study design, type of exposure or participants, altitude, study quality, or severity of AMS. However, we could not use the adjusted OR for the meta-analysis because most authors did not adjust for smoking in the original manuscripts. Therefore, these data were not available. However, evaluation of cigarettes per day might lend support for an association between exposure and disease [25]. Therefore, further investigation into the role of smoking in AMS risk is needed, with an emphasis on smoking duration and the number of cigarettes smoked. Heterogeneity is often a concern in a meta-analysis. Evidence of substantial heterogeneity across studies of the associations of smoking and AMS risk was observed in our meta-analysis, primarily in high-quality cohort studies, high altitudes, and mountaineer populations. Smoking and AMS definitions without sample size estimations differed in across studies, which affected prospective studies because the power to confirm the hypothesis was suboptimal. Moreover, the included studies were conducted in different countries and on different continents, and these populations likely have different smoking prevalence, lifestyle, prevention measures, and AMS incidence. Therefore, the characteristics of subjects and study design might have contributed to the observed heterogeneity. The subgroup analyses of cross-sectional or case-control studies, low altitudes, moderate quality observational studies, and mountaineers detected no significant heterogeneity (*P* > 0.05). These other studies likely did not estimate a formal sample size, but the feasibility to develop these study designs in a short period was demonstrated. The same authors developed some of them, and heterogeneity was almost absent.

### 4.2. Altitude

We ignored the specific ascent rate in each study; hence, we considered only the maximum altitude the participants reached. Low altitude was defined when the highest altitude reached was below 3500 MASL; moderate to high altitude occurred when the greatest altitude was equal to or higher than 3500 MASL. No significant reduction in AMS risk was associated with low or high altitude in smokers. Some authors found that smoking protected against AMS development [11, 14], whereas others found that smoking increased AMS development [12, 13] in different altitudes.

### 4.3. Mountaineers

The effect of the type of participants was based on the likely presence and absence of training in mountaineers and nonmountaineers, respectively. Most evaluated studies did not provide stratified data of AMS by smoking status or previous physical training (e.g., the number of nights spent above 3000 MASL or months before the ascent). Previous physical training could produce some impact on acclimatisation. Therefore, the decrease in AMS incidence could be quite similar to the smoking effect on acclimatisation. However, this effect was not demonstrated in the subgroup analyses because mountaineers frequently ascend to higher altitudes, and this group showed previous training and a low smoking prevalence. Mountaineers showed a low smoking prevalence (11.3%) in our subgroup analysis, and nonmountaineers exhibited a higher prevalence (55.5%) (*P* < 0.001). These findings could be due to a comparison between mountaineers, who go to higher altitudes, and nonmountaineers.

### 4.4. Limitations

First, the observed association may be masked by the lack of temporality because our analysis was primarily based on cross-sectional studies. These studies were developed in huts just prior to ascent. Therefore, the development of AMS that was described by the authors as prevalent episodes should likely be described as incident events. Smoking status was described at the time of AMS development, but smoking was obviously present before the ascent. Moreover, unmeasured or residual confounding is always a major concern in observational studies. The results of sensitivity and subgroup analyses showed robustness, and the relationship between AMS and smoking was not influenced by study design or quality, altitude or the type of participant or exposure. Therefore, the likelihood that our findings resulted from other unmeasured confounders cannot be excluded, and our meta-analysis suffers from heterogeneity and lack of studies that specifically looked at smoking and AMS, so more studies are needed. Second, we were unable to evaluate the dose–response effect of smoking on AMS development because it was not described in any study. Third, the AMS case definition was based on different diagnosis criteria or questionnaires. Headache is a cornerstone symptom for the diagnosis of AMS in Western countries; however, in China, it is not essential, and some language discrepancies between LLS and the Chinese Scoring System make difficult their direct comparison. Finally, one study defined AMS according to compression chamber use, so this study probably underestimated the mild AMS prevalence. Therefore, a misclassification of subjects was possible, and the relationship between smoking and AMS risk may be under- or overestimated. Finally, publication bias may be a problem because studies with null effects are less likely to be published than studies that provide statistically significant results. Egger's test and Begg's funnel plots detected no evidence of publication bias in the meta-analysis, but that estimation may not be sufficiently accurate because the number of included studies and population was relatively small.

## 5. Conclusions

In summary, the present meta-analysis of 11 observational studies indicated that smoking did not either reduce or increase the risk of AMS. However, many questions must be addressed. Further large-scale prospective studies, using a strict case definition of AMS and smokers and confounders, are warranted to validate our findings.

## Figures and Tables

**Table 1:** Characteristics of included studies according to Newcastle-Ottawa Quality Assessment Scale.

Study	Selection	Comparability	Exposure/ outcome	NOS scale
Wu et al., 2012	⋆⋆⋆	⋆	⋆⋆	6
Li et al., 2011	⋆⋆⋆	⋆⋆	⋆⋆	7
Wagner et al., 2008	⋆⋆⋆	⋆	⋆	5
You et al., 2012	⋆⋆⋆	⋆⋆	⋆⋆	7
Vinnikov et al., 2014	⋆⋆⋆	⋆	⋆⋆	6
McDevitt et al., 2014	⋆⋆⋆	⋆	⋆	5
Mairer et al., 2009	⋆⋆⋆	⋆	⋆	5
Mairer et al., 2010	⋆⋆⋆	⋆	⋆	5
Mairer et al., 2010	⋆⋆⋆	⋆	⋆	5
Schneider et al., 2001	⋆⋆⋆	⋆	⋆	5
Schneider et al., 2001	⋆⋆⋆	⋆	⋆	5

*Note*. Each star represents a high-quality criterion accomplished by the study.

**Table 2:** Characteristics of included studies.

Study	Final altitude (MASL)	Design	*n*	Smoking	AMS	Smoking adjustment
Wu et al., 2012	4552 *MASL* (4292–4905 *MASL*), Qinghai-Tibet railroad; occupational cohort	Cohort study	382	Smoking status: (i) No smoker (ii) Smoker < 20 cig/d (iii) Smoker 20 cig/d (iv) Smoker > 20 cig/d	LLS ≥ 4	Absent
Li et al., 2011	(2900–4300 *MASL*); Qinghai Tibet plateau; military cohort	Cohort study	3727	Smoking status: (i) No current smoker (ii) Current smoker	SSQ	Present
Wagner et al., 2008	4419 *MASL* (2550–4419) Mt. Whitney, California, USA; Hikers	Cross-sectional	886	Smoking status: (i) Smoker (ii) Nonsmoker	LLS ≥ 3	Absent
You et al., 2012	4300 *MASL* (2860–5250 *MASL*); Military cohort	Cohort study	314	Smoking status: (i) Not current smoker (ii) Current smoker	LLS ≥ 4	Present
Vinnikov et al., 2014	4000 *MASL* (3800–4200 *MASL*) Tyan Shan Mountains, Kyrgyzstan; Occupational cohort	Nested case-control (1 : 2)	45	Smoking status: (i) Smoker <10 cig/d (ii) Smoker ≥10 cig/d (iii) Nonsmoker	SSQ	Absent
McDevitt et al., 2014	(3500–5400 *MASL*); Thorong-La, Annapurna, Nepal; Trekkers	Cross-sectional	332	Smoking status: (i) Smoker (ii) Nonsmoker	LLS ≥ 3 LLS ≥ 5 ESQ > 0.7	Present
Mairer et al., 2009	2200–3500 *MASL* Eastern Alps; Mountaineers	Cross-sectional	431	Smoking status: (i) Smoker (ii) Nonsmoker	LLS ≥ 4	Absent
Mairer et al., 2010	3454-4049 *MASL* in Eastern Alps; Mountaineers	Cross-sectional	79	Smoking status: (i) Smoker (ii) Nonsmoker	LLS ≥ 4	Absent
Mairer et al., 2010	3817-4808 *MASL* in Western Alps; Mountaineers	Cross-sectional	83	Smoking status: (i) Smoker (ii) Nonsmoker	LLS ≥ 4	Absent
Schneider et al., 2001	4559 *MASL*; Capanna Margherita, Italy; Mountaineers in 1996 and 1999	Cross-sectional	440	Smoking status: (i) No smoking (ii) Smoking	ESQ > 0.7	Present
Schneider et al., 2001	4559 *MASL*; Capanna Margherita, Italy; Mountaineers in 2000	Cross-sectional	387	Smoking status: (i) No smoking (ii) Smoking	ESQ > 0.7	Present

SSQ: study-specific questionnaire; AMS: acute mountain sickness; ESQ: environmental symptom questionnaire; NS: not stated; LLS: Lake Louise Score.

**Table 3:** Subgroup analyses of RRs for the association between AMS and smoking.

Group	Number of studies	Pooled RR (95% CI)	Heterogeneity	
			*I* ^2^	*P*
Overall	11	1.02 (0.83, 1.26)	73.0%	<0.001
Study design				
Cross-sectional studies and case-control study	8	1.20 (0.99, 1.47)	32.0%	0.169
Cohort studies	3	0.79 (0.54, 1.17)	89.0%	<0.001
Altitude				
<3500 MASL	2	1.24 (0.78, 1.95)	25.0%	0.922
≥3500 MASL	9	1.00 (0.79, 1.26)	78.0%	<0.001
Quality assessment				
NOS/aNOS = 5	7	1.17 (0.97, 1.41)	23.0%	0.253
NOS/aNOS = 6-7	4	0.92 (0.62, 1.36)	87.0%	<0.001
Type of participant				
Mountaineer	7	1.17 (0.97, 1.41)	23.0%	0.253
Nonmountaineer	4	0.92 (0.62, 1.36)	87.0%	<0.001

AMS: acute mountain sickness; CI: confidence interval; MASL: meters above sea level; NOS: Newcastle-Ottawa Quality Assessment Scale; aNOS: adapted Newcastle-Ottawa Quality Assessment Scale; RR: relative risks. Relative risks were obtained using the DerSimonian and Laird random-effect model.

**Table 4:** Results of sensitivity analyses for AMS risk by smoking status.

ID	Study omitted	Pooled RR (95% CI)	Heterogeneity	
			*I* ^2^	*P*
(1)	Wu et al., 2012	1.07 (0.85, 1.34)	70.0%	<0.001
(2)	Li et al., 2011	1.02 (0.78, 1.32)	76.0%	<0.001
(3)	Wagner et al., 2008	1.01 (0.80, 1.27)	73.0%	<0.001
(4)	You et al., 2012	1.10 (0.92, 1.32)	59.0%	0.002
(5)	Vinnikov et al., 2014	0.98 (0.80, 1.21)	73.0%	<0.001
(6)	McDevitt et al., 2014	0.97 (0.78, 1.19)	67.0%	<0.001
(7)	Mairer et al., 2009	1.01 (0.81, 1.26)	76.0%	<0.001
(8)	Mairer et al., 2010 (Eastern Alps)	1.02 (0.82, 1.27)	76.0%	<0.001
(9)	Mairer et al., 2010 (Western Alps)	1.04 (0.84, 1.29)	75.0%	<0.001
(10)	Schneider et al., 2001 (sub-study 1)	1.02 (0.81, 1.28)	76.0%	<0.001
(11)	Schneider et al., 2001 (sub-study 2)	1.04 (0.83, 1.31)	75.0%	<0.001

AMS: acute mountain sickness; CI: confidence interval; RR: relative risks. Relative risks were obtained using the DerSimonian and Laird random-effect model.

## References

[1] Hackett P. H., Roach R. C., Auerbach P. S. (2012). High-altitude medicine and physiology. *Wilderness Medicine*.

[2] Stobdan T., Karar J., Pasha M. A. Q. (2008). High altitude adaptation: genetic perspectives. *High Altitude Medicine and Biology*.

[3] Vardy J., Judge K. (2006). Acute mountain sickness and ascent rates in trekkers above 2500 m in the Nepali Himalaya. *Aviation Space and Environmental Medicine*.

[4] Schneider M., Bernasch D., Weymann J., Holle R., Bartsch P. (2002). Acute mountain sickness: influence of susceptibility, preexposure, and ascent rate. *Medicine and Science in Sports and Exercise*.

[5] Karinen H., Peltonen J., Tikkanen H. (2008). Prevalence of acute mountain sickness among finnish trekkers on Mount Kilimanjaro, Tanzania: an observational study. *High Altitude Medicine and Biology*.

[6] Askew E. W. (2004). Food for high-altitude expeditions: Pugh got it right in 1954. *Wilderness and Environmental Medicine*.

[7] Nerín M. A., Palop J., Montaño J. A., Morandeira J. R., Vázquez M. (2006). Acute mountain sickness: influence of fluid intake. *Wilderness and Environmental Medicine*.

[8] Basnyat B., Gertsch J. H., Holck P. S. (2006). Acetazolamide 125 mg BD is not significantly different from 375 mg BD in the prevention of acute mountain sickness: the prophylactic acetazolamide dosage comparison for efficacy (PACE) trial. *High Altitude Medicine and Biology*.

[9] West R., Hajek P., Stead L., Stapleton J. (2005). Outcome criteria in smoking cessation trials: proposal for a common standard. *Addiction*.

[10] Mertens H. M., Mannebach H., Gleichmann U. (1979). Non-invasive effects of cigarette smoking on left ventricular function at rest and with exercise in normal individuals. *Zeitschrift fur Kardiologie*.

[11] Wu T.-Y., Ding S.-Q., Liu J.-L. (2012). Smoking, acute mountain sickness and altitude acclimatisation: a cohort study. *Thorax*.

[12] Vinnikov D., Brimkulov N., Krasotski V., Redding-Jones R., Blanc P. D. (2014). Risk factors for occupational acute mountain sickness. *Occupational Medicine (Oxford, England)*.

[13] McDevitt M., McIntosh S. E., Rodway G., Peelay J., Adams D. L., Kayser B. (2014). Risk determinants of acute mountain sickness in trekkers in the Nepali Himalaya: a 24-year follow-up. *Wilderness and Environmental Medicine*.

[14] You H., Li X., Pei T., Huang Q., Liu F., Gao Y. (2012). Predictive value of basal exhaled nitric oxide and carbon monoxide for acute mountain sickness. *Wilderness and Environmental Medicine*.

[15] Li X., Tao F., Pei T., You H., Liu Y., Gao Y. (2011). Population level determinants of acute mountain sickness among young men: a retrospective study. *BMC Public Health*.

[16] Wagner D. R., D'Zatko K., Tatsugawa K. (2008). Mt. Whitney: determinants of summit success and acute mountain sickness. *Medicine and Science in Sports and Exercise*.

[17] Mairer K., Wille M., Bucher T., Burtscher M. (2009). Prevalence of acute mountain sickness in the eastern Alps. *High Altitude Medicine and Biology*.

[18] Mairer K., Wille M., Burtscher M. (2010). The prevalence of and risk factors for acute mountain sickness in the Eastern and Western Alps. *High Altitude Medicine and Biology*.

[19] Liberati A., Altman D. G., Tetzlaff J. (2009). The PRISMA statement for reporting systematic reviews and meta-analyses of studies that evaluate healthcare interventions: explanation and elaboration. *The British Medical Journal*.

[20] Wells G., Shea B., OConnell D. (2009). *The Newcastle-Ottawa Scale (NOS) for assessing the quality of nonrandomised studies in meta-analyses*.

[21] Greenland S. (1987). Quantitative methods in the review of epidemiologic literature. *Epidemiologic Reviews*.

[22] Sampson J. B., Cymerman A., Burse R. L., Maher J. T., Rock P. B. (1983). Procedures for the measurement of acute mountain sickness. *Aviation Space and Environmental Medicine*.

[23] Imray C., Booth A., Wright A., Bradwell A. (2011). Acute altitude illnesses. *BMJ*.

[24] West J. B. (2010). English translation of "nomenclature, classification, and diagnostic criteria of high altitude disease in china". *High Altitude Medicine and Biology*.

[25] Hamling J., Lee P., Weitkunat R., Ambühl M. (2008). Facilitating meta-analyses by deriving relative effect and precision estimates for alternative comparisons from a set of estimates presented by exposure level or disease category. *Statistics in Medicine*.

[26] Waeber B., Kayser B., Dumont L., Lysakowski C., Tramèr M. R., Elia N. (2015). Impact of study design on reported incidences of acute mountain sickness: a systematic review. *High Altitude Medicine and Biology*.

[27] DerSimonian R., Laird N. (1986). Meta-analysis in clinical trials. *Controlled Clinical Trials*.

[28] Higgins J. P. T., Thompson S. G. (2002). Quantifying heterogeneity in a meta-analysis. *Statistics in Medicine*.

[29] Higgins J. P. T., Thompson S. G., Deeks J. J., Altman D. G. (2003). Measuring inconsistency in meta-analyses. *British Medical Journal*.

[30] Egger M., Smith G. D., Schneider M., Minder C. (1997). Bias in meta-analysis detected by a simple, graphical test. *British Medical Journal*.

[31] Begg C. B., Mazumdar M. (1994). Operating characteristics of a rank correlation test for publication bias. *Biometrics*.

[32] Duval S., Tweedie R. (1988). Practical estimates of the effect of publication bias in meta-analysis. *Australasian Epidemiologist*.

[33] Vinnikov D., Blanc P. D., Steinmaus C. (2016). Is smoking a predictor for acute mountain sickness? findings from a meta-analysis. *Nicotine and Tobacco Research*.

[34] Xu C., Lu H. X., Wang Y. X., Chen Y., Yang S. H., Luo Y. J. (2016). Association between smoking and the risk of acute mountain sickness: a meta-analysis of observational studies. *Military Medical Research*.

